# Protocol to define the *in situ* proteome of endogenous PRC2 bodies in the triple-negative breast cancer cell line BoM-1833 using optoproteomics

**DOI:** 10.1016/j.xpro.2026.104578

**Published:** 2026-05-14

**Authors:** Yu-Chih Pan, Hsiao-Jen Chang, Weng Man Chong, Jung-Chi Liao, Cristiana Lungu

**Affiliations:** 1 Institute of Cell Biology and Immunology, University of Stuttgart, Stuttgart 70569, Germany; 2 Stuttgart Research Center Systems Biology, University of Stuttgart, Stuttgart 70569, Germany; 3 Syncell Inc., Taipei 115202, Taiwan

**Keywords:** Cell Biology, Cancer, Proteomics

## Abstract

Polycomb repressive complex 2 (PRC2) and its catalytic subunit EZH2 regulate transcriptional repression and display subcellular organization patterns important for cell function. Here, we present a protocol that applies image-guided optoproteomics to profile the *in situ* proteome of endogenous EZH2-containing PRC2 bodies in the triple-negative breast cancer cell line BoM-1833. We describe steps for cell culture, EZH2 immunofluorescence, and Microscoop-based photolabeling. We then detail procedures for protein enrichment and mass spectrometry for profiling chromatin-associated signaling.

For complete details on the use and execution of this protocol, please refer to Pelzer et al.^1^

## Highlights

• Steps for immunostaining of BoM-1833 cells to preserve endogenous EZH2/PRC2 bodies
• Guidance for photo-biotinylation of EZH2 bodies with Microscoop for optoproteomics
• Steps for enriching biotinylated proteins and mapping EZH2 body proteome by LC-MS/MS

## Abstract (editor highlights)

Publisher’s note: Undertaking any experimental protocol requires adherence to local institutional guidelines for laboratory safety and ethics.

## Graphical abstract

**Figure:**
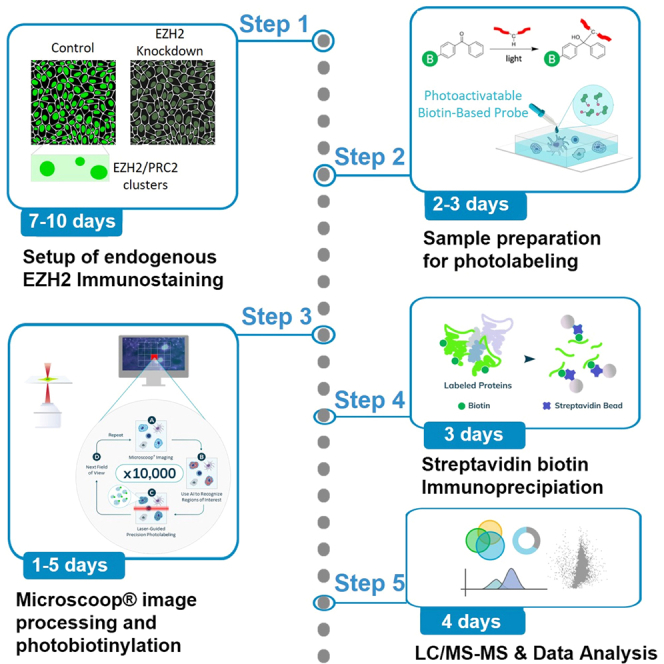

## Before you begin

Chromatin is organized into spatially defined subnuclear compartments that contribute to the regulation of gene expression programs and^1^ cellular identity states.^2^ Furthermore, chromatin regulators themselves, such as PRC2, can form optically resolved nuclear subcomparments, commonly referred to as Polycomb bodies^3^^,^^4^ whose local proteome can influence chromatin states and signaling pathways. Defining the in-situ proteome of these specialized compartments requires high-resolution imaging and precise, spatially targeted labeling.^1^

This protocol involves extensive mammalian cell culture techniques. Users should have access to specialized tissue culture facilities, including a certified biosafety cabinet, CO_2_-regulated 37°C incubators, inverted microscopes, and standard cell culture instrumentation. In addition, the protocol requires confocal imaging for EZH2 cluster visualization and optoproteomics instrumentation (i.e., Microscoop® Mint) for photolabeling. Downstream protein enrichment and identification require streptavidin-based pull-down and liquid chromatography–tandem mass spectrometry (LC–MS/MS) capabilities.

Familiarity with immunofluorescence staining, siRNA transfection, and basic data analysis workflows is recommended.

### Innovation

This protocol presents an optimized spatial proteomics workflow that applies Syncell’s Microscoop® technology to define the *in-situ* proteome of endogenous PRC2 bodies in the triple negative (TNBC) cell line BoM-1833. Building on the proof-of-principle EZH2-cluster optoproteomics study by Pelzer et al.,^1^ the protocol provides a bench-ready implementation for analyzing visually defined EZH2 positive Polycomb compartments in BoM-1833 cells. Traditional biochemical or proximity labeling approaches lack the resolution to isolate submicron, visually defined chromatin compartments and cannot distinguish local proteomes from the surrounding nucleoplasm.^5^^,^^6^ In contrast, Microscoop®enables fluorescence-guided region-of-interest selection and two-photon photolabeling with submicron precision, allowing selective tagging of proteins within EZH2-marked PRC2 bodies directly inside fixed, intact nuclei.^1^

The protocol merges high-resolution immunofluorescence imaging, siRNA-validated EZH2 detection, and precision optoproteomics into a unified workflow optimized for epithelial cancer cells. By combining automated image analysis, targeted photolabeling, and streptavidin-based enrichment with downstream LC–MS/MS, this approach enables unbiased identification of factors that concentrate within endogenous, optically resolvable PRC2 compartments without the need for genetic tagging, overexpression, or large-scale chromatin fractionation.

Together, this protocol demonstrates the utility of Microscoop® to epigenetic protein complexes and provides a workflow for spatially resolved proteomic analysis of EZH2 -associated regulatory networks.

### Culturing and maintenance of the BoM-1833 cell line

**Timing: 3–4 days**

The following steps describe how to culture and maintain the BoM-1833 cell line, a human TNBC cell line derived by the lab of Joan Massagué from bone metastases following cardiac inoculation of nude mice with the parental MDA-MB-231 line.^7^

1. Remove medium from culture dish by gentle aspiration.
2. Wash cells once with sterile Ca^2+^/Mg^2+^ - free 1× PBS.
3. Add enough trypsin-EDTA to completely cover the cells.
4. Place the dish at 37°C for 1 to 2 min.
5. Check under the microscope if the cells have detached from the plate.

**CRITICAL:** BoM-1833 cells detach easily and are sensitive to trypsin, especially when not fully confluent. It is however important to ensure that all cells have detached before proceeding to the next step.

6. Neutralize trypsin by adding 4 volumes of complete culture medium per 1 volume of trypsin–EDTA.
7. Collect all the liquid in a sterile tube.
8. Centrifuge the cell suspension for 3 min at 500 *g* at 20°C–25°C.
9. Remove the supernatant by aspiration.
10. Mix the cell pellet with fresh complete medium.
11. Determine cell concentration using a hemocytometer or an automatic cell counting device.

***Note:*** In this step you can also check cell viability, which should be higher than 95%.

**CRITICAL:** Avoid splitting BoM-1833 cells at ratios greater than 1:6, as this can lead to reduced growth rates. We routinely seed 3 × 10^6^ cells into a fresh T75 flask and passage the cells every 2–3 days.

## Key resources table

**Table:** 

REAGENT or RESOURCE	SOURCE	IDENTIFIER
**Antibodies**		

rabbit anti-EZH2	Cell Signaling Technology	Cat# 5246; RRID:AB_10694683 1: 250 for regular immunofluorescence 1: 500 for optoproteomics 1: 1000 for western blot
Goat anti-Rabbit IgG (H+L) Cross-Adsorbed Secondary Antibody, Alexa Fluor™ 488	Thermo Fisher Scientific	Cat# A-11008; RRID:AB_143165 1:2500
Goat anti-Rabbit IgG (H+L) Highly Cross-Adsorbed Secondary Antibody, Alexa Fluor™ 647	Thermo Fisher Scientific	Cat# A-21245; RRID:AB_2535813 1:2500

**Chemicals, peptides, and recombinant proteins**		

RPMI 1640 medium	ThermoFisher Scientific	Cat# 11875093
FBS	ThermoFisher Scientific; Gibco	Cat# A52567-01
Optimem	ThermoFisher Scientific; Gibco	Cat# 31985-047
Pen/Strep	ThermoFisher Scientific; Gibco	Cat# 15140-122
1× PBS, No Calcium and Magnesium	ThermoFisher Scientific; Gibco	Cat# 10010023
1× PBS, With Calcium and Magnesium	ThermoFisher Scientific; Gibco	Cat# 14040133
10× PBS, With Calcium and Magnesium	ThermoFisher Scientific; Gibco	Cat# 14080055
TrypLE™ Select Enzyme (10x) for cell culture	ThermoFisher Scientific; Gibco	Cat# A1217701
4′,6-Diamidino-2-phenylindole dihydrochloride (=DAPI)	Merck	Cat# D8417-5MG
Phalloidin Alexa Fluor™ 647	ThermoFisher Scientific	Cat# A22287
ProLong™ Gold Antifade Mountant	ThermoFisher Scientific	Cat# P10144
BSA Fraction V	Carl Roth	Cat# 8076.2
Paraformaldehyde	Merck	Cat # 158127-5G
Natriumhydroxid	Carl Roth	Cat # P031.1
Hydrochloric acid 37%	Carl Roth	Cat # 4625.1
Collagen R solution 0.2 %	Serva	Cat # 47254.01
Triton® X 100	Carl Roth	Cat# 3051.1
Formic acid (≥95%)	Merck	Cat# F0507
Formic Acid Solution (0.1% (v/v) Formic acid in water)	Merck	Cat # 1.59013
Acetonitrile (≥99.9%)	Merck	Cat # 1.00030
Acetonitrile solution (contains 0.1% (v/v) formic acid)	Merck	Cat # 1.59002
Lipofectamine™ RNAiMAX Transfection reagent	ThermoFisher Scientific	Cat# 13778075
Sodium azide	Merck	Cat# S2002-5G
Pierce™ 660 nm Protein-Assay-Kit	ThermoFisher Scientific	Cat# 22662
Ionic Detergent Compatibility Reagent for Pierce™ 660nm Protein Assay Reagent	ThermoFisher Scientific	Cat# 22663

**Critical commercial assays**		

Synlight-Rich™ kit	Syncell, Taiwan	Cat# SYN-RI0106
Synpull™ reagent kit	Syncell, Taiwan	Cat# SYN-PU0106
One-well chamber slide for photolabeling	Cellvis, USA	Cat# C1-1.5H-N

**Deposited data**		

Proteomics EZH2 dataset	Pelzer et al. 2025^1^	ProteomeXchage: PXD068214; MassIVE: MSV000099095

**Experimental models: Cell lines**		

BoM-1833	Kindly provided by Joan Massague’, Memorial Sloan Kettering Cancer Center, USA	RRID: CVCL_DP48

**Oligonucleotides**		

siNT, ON-TARGETplus non-targeting control pool	Dharmacon	Cat# D-001810-10
siEZH2 Silencer Select	ThermoFisher Scientific	Cat# s4917

**Software and algorithms**		

Zeiss ZEN 3.3	https://www.zeiss.com/microscopy/en/products/software/zeiss-zen.html	N/A
Proteome Discoverer version 2.4.1.15	Thermo Fisher Scientific	https://www.thermofisher.com/tw/en/home/industrial/mass-spectrometry/liquid-chromatography-mass-spectrometry-lc-ms/lc-ms-software/multi-omics-data-analysis/proteome-discoverer-software.html/
Autoscoop (1.1.0.5)	Syncell	https://www.syncell.com/microscoop/
ImageJ	NIH	https://imagej.net/ij/

**Other**		

LSM980 Airyscan 2	Carl Zeiss, Oberkochen, Germany	N/A
Microscoop Mint (MS.1012)	Syncell	https://www.syncell.com/microscoop/
UltiMate 3000 RSLCnano	Thermo Fisher Scientific	https://www.thermofisher.com/
Orbitrap Fusion Lumos	Thermo Fisher Scientific	https://www.thermofisher.com/
ZipTip with 0.6 μL C18 resin	Merck Millipore	Cat# ZTC18S096

## Materials and equipment

**Table:** Complete cell culture medium: Supplemented RPMI

Reagent	Final concentration	Amount
Roswell Park Memorial Institute (RPMI) 1640 Medium	N/A	450 mL
Fetal bovine serum (FBS)	10%	50 mL
**Total**	**N/A**	**500 mL**

Storage conditions: 4°C, up to 4 weeks.

### 2.4% PFA

Add 12 g of paraformaldehyde (PFA) to 100 mL ddH_2_O. Add 200 μL 2 N NaOH. Stir mixture on hot plate at 65°C until PFA is dissolved. Once the solution is clear, add 50 mL 10× PBS and 350 mL ddH_2_O. Stir mixture, allow to cool down at 20°C–25°C and check pH. The pH should be 7.2–7.3. If pH is too high, adjust pH by adding 37% HCl (w/v) dropwise.

Store in 10 mL aliquots at −20°C for several months.

***Note:*** To ensure reproducible fixation and avoid degraded reagent affecting sample quality, thaw the PFA aliquots on the day of use and discard the remaining solution.

**CRITICAL:** PFA is highly toxic and should be prepared in a chemical hood. NaOH and HCl are corrosive. PFA should always be handled in a fume hood. Wear laboratory coat, gloves and goggles when working with PFA, NaOH and HCl.

**Table:** Permeabilization buffer 0.5% Triton

Reagent	Final concentration	Amount
PBS	1×	95.5 mL
Triton X-100	0.5%	0.5 mL
**Total**	**N/A**	**100 mL**

Storage conditions: 4°C, up to 2 weeks. Equilibrate an aliquot at RT before usage.

**Table:** Blocking solution for immunofluorescence

Reagent	Final concentration	Amount
PBS	1×	100 mL
BSA	3%	3 g
**Total**	**N/A**	**100 mL**

Sterilize with a 0.22-μm filter. Storage conditions: 4°C, up to 4 weeks.

### Block 1 buffer (Synlight-Rich kit)

Vortex Block 1 (A) at 500 rpm for 5 min at 20°C–25°C, and spin down briefly to collect contents at the bottom of the tube. For each biological replicate of the optoproteomics experiment, two groups of samples are prepared: one group is exposed to light under the Microscoop for photo-biotinylation (Photolabeled, PL); the other group is kept in the dark and serves as an unlabeled control (UL). For each pair of cell samples (1 photolabeled sample PL and 1 unlabeled control UL), freshly prepare Block 1 Buffer before use by mixing 40 μL of Block 1 (A) with 1960 μL 1× PBS containing 0.1% Triton X-100.

### Block 2 buffer (Synlight-Rich kit)

Vortex Block 2 (B) at 500 rpm for 5 min at 20°C–25°C, and spin down briefly to collect contents.

For each pair of cell samples (1 PL, 1UL), freshly prepare Block 2 Buffer before use by mixing 40 μL of Block 2 (B) with 1960 μL 1× PBS containing 0.1% Triton X-100.

### Labeling reagent (Synlight-Rich kit)

Vortex Photolabel (C) at 500 rpm for 10 min at 20°C–25°C, and spin down briefly to collect contents.

For each pair of samples (1 PL, 1UL), freshly prepare Labeling Reagent before use by mixing 40 μL of Photolabel (C) with 1960 μL 1× PBS.

**CRITICAL:** Prepare Labeling Reagent in a light-controlled room. Ensure the lighting is at a wavelength of > 500 nm to prevent undesirable reactions. In our laboratory, procedures were performed in a dedicated red-light room.

### Quencher (Synlight-Rich kit)

Vortex Quench (D) at 500 rpm for 5 min at 20°C–25°C, and spin down briefly to collect contents.

For each pair of cell samples (1 PL, 1UL), freshly prepare Quencher before use by mixing 120 μL of Quench (D) with 5.88 mL 1× PBS containing 0.1% Triton X-100.

### Verification buffer (Synlight-Rich kit)

Vortex Verify (G) and spin down briefly to collect contents.

For one PL sample, freshly prepare Verification Buffer before use by mixing 20 μL of Verify (G) with 980 mL 3% BSA in 1× PBS containing 0.1% Triton X-100.

### Solution B (lyse 1) (Synpull reagent kit)

Spin down B (Lyse 1), add 50 μL of ultrapure water, and vortex it vigorously until the powder is fully dissolved.

***Note:*** Store the tube at −20°C after use until expiration date and avoid exceeding three freeze-thaw cycles.

### Lysis buffer (Synpull reagent kit)

Vortex C (Lyse 2) at 500 rpm at 20°C–25°C until it appears as a clear solution.

For each pair of cell samples (1 PL, 1UL), freshly prepare Lysis Buffer before use by combining 15 μL of Solution B (Lyse 1) and 117 μL of C (Lyse 2).

### Solution D (lyse 3) (Synpull reagent kit)

Spin down D (Lyse 3), add 100 μL of ultrapure water, then vortex it vigorously until the powder is fully dissolved.

***Note:*** Store the tube at −20°C after use until expiration date and avoid exceeding three freeze-thaw cycles.

### Solution K (digest) (Synpull reagent kit)

Spin down K (Digest), add 150 μL of J (Wash 4) to the tube, and vortex at 500 rpm for 5 min at 20°C–25°C. Keep the solution at 4°C before use.

***Note:*** Store the tube at −20°C after use until expiration date and avoid exceeding three freeze-thaw cycles.

**Table:** Desalt 1 buffer

Reagent	Final concentration	Amount
Acetonitrile (≥99.9%)	90%	45 mL
ddH_2_O	N/A	5 mL
**Total**	**N/A**	**50 mL**

Storage conditions: 20-25°C, up to 4 weeks.

**Table:** Desalt 2 buffer

Reagent	Final concentration	Amount
Formic acid (≥95%)	0.1%	50 μL
ddH_2_O	N/A	49.95 mL
**Total**	**N/A**	**50 mL**

Storage conditions: 20-25°C, up to 4 weeks.

**Table:** Desalt 3 buffer

Reagent	Final concentration	Amount
Acetonitrile (≥99.9%)	80%	40 mL
Formic acid (≥95%)	0.1%	50 μL
ddH_2_O	N/A	9.95 mL
**Total**	**N/A**	**50 mL**

Storage conditions: 20-25°C, up to 4 weeks.

## Step-by-step method details

### Setup of endogenous EZH2 immunofluorescence conditions

**Timing: 7–10 days**

Workflow for immunofluorescent detection of endogenous EZH2 in BoM-1833 cells, including antibody specificity validation using siEZH2 knockdowns. EZH2 is used here as a proxy for the PRC2 complex.

***Note:*** While we have used BoM-1833 cells for this protocol, users can replicate the workflow in any cell line that displays PRC2 clusters visible by confocal microscopy. We observed that the TNBC cell line MDA-MB-436 forms prominent PRC2 bodies. In addition, we showed that overexpression of GFP-PHF19 for 24 h is sufficient to induce de novo PRC2 clusters in BT549, HS578T, and MCF7 cells.^1^ Endogenous PRC2 bodies have also been described in mouse embryonic stem cells cultured in 2i medium.^8^

**Figure 1:**
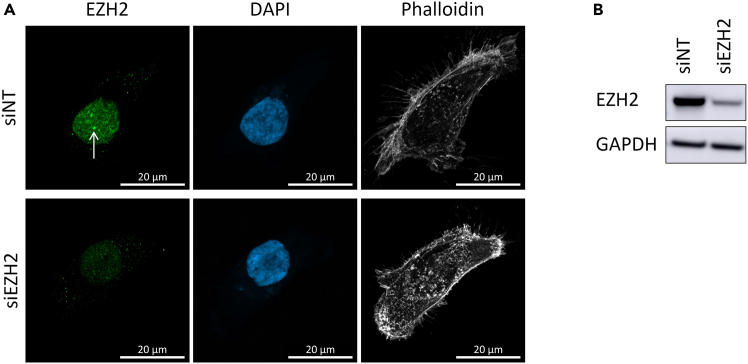
Validation of the EZH2 immunofluorescence antibody in BoM-1833 cells (A) BoM-1833 cells were transfected with EZH2-targeting siRNA or a non-targeting control (siNT) to assess antibody specificity. Representative confocal microscopy images show cells fixed 72 h post-transfection and immunostained for endogenous EZH2 (green). Nuclei and F-actin were counterstained with DAPI (blue) and phalloidin (white), respectively. Loss of EZH2 signal upon EZH2 knockdown confirms antibody specificity. The arrow indicates a representative PRC2 cluster. Images were acquired and displayed using identical settings. Scale bar, 20 μm. (B) BoM-1833 cells transfected as in (A) were analyzed by western blot with GAPDH as loading control.

**CRITICAL:** Antibody validation is essential for interpreting EZH2 localization and for meaningful downstream isolation of the local PRC2 body proteome. Only antibodies and immunofluorescence conditions that (1) show a strong reduction of EZH2 signal in siEZH2-treated cells and (2) produce optically defined EZH2 clusters in control cells should be used for subsequent optoproteomics (Figure 1). See also troubleshooting 1 and 2.

***Note:*** For targets for which no reliable immunofluorescence antibody is available, fluorescently tagged protein constructs can be used as an alternative. Be sure to confirm that the tag does not alter the protein’s expression level, localization, or clustering behavior.

1. Perform reverse transfection of BoM-1833 cells.
  a. Thaw siRNA stocks on ice.
  b. Prepare the siRNA mixture as described below. Prepare one mixture for non-targeting control siRNA (siNT) and one for siEZH2.

**Table:** 

Reagent	Final concentration	Amount
Optimem	N/A	500 μL
siRNA stock, 20 μM	5 nM	1.5 μL
Lipofectamine RNAiMAX Transfection Reagent	N/A	10 μL
**Total**	**N/A**	**511.5 μL**
  c. Vortex briefly and incubate siRNA mixtures for 15–20 min at 20°C–25°C.
  d. Prepare a BoM-1833 cell suspension in complete growth medium at 3 × 10^5^ cells/mL.
  e. Distribute 5 mL of the cell suspension in a 6-cm dish (one dish per siRNA condition).
  f. Add the siRNA transfection mixture dropwise and gently shake the dish back and forth to distribute.
  g. Place the dishes for further 48 h in the 37°C cell culture incubator.
    ***Note:*** Reverse transfection ensures uniform siRNA delivery and yields robust EZH2 knockdown required for antibody validation.
2. BoM-1833 cell seeding on glass coverslips for immunofluorescence staining.
  a. Prepare collagen-coated coverslips.
    i. Place sterile glass coverslips into a 12-well plate.
    ii. Add 1 mL 10 μg/mL collagen/PBS solution to each coverslip.
      ***Note:*** Ensure that the coverslips remain fully submerged in the collagen solution during coating. Use a sterile pipette tip to gently press them down if they float.
    iii. Allow the collagen to polymerase for 2 h at 37°C.
    iv. Aspirate collagen solution and rinse twice with sterile PBS.
    v. Store glass coverslips in cell culture media at 37°C until cell seeding.
      **CRITICAL:** Collagen coating improves epithelial cell adhesion and preserves morphology during fixation.
      ***Note:*** Coverslips can also be pre-coated 3–5 days before. In this case, collagen polymerization can occur overnight at 4°C. Before use, equilibrate coated coverslips in culture medium for at least 30 min at 37°C to ensure proper cell attachment.
  b. Cell seeding on collagen-coated coverslips.
    i. For each siRNA knockdown transfection, prepare a cell suspension of 1.5 × 10^5^ cells/mL.
    ii. Seed 1 mL cell suspension per coverslip.
    iii. Incubate the cells overnight in the 37°C cell culture incubator.
      ***Note:*** Retain an aliquot of the cell suspension to independently validate siRNA knockdown efficiency (e.g., by Western blotting or qPCR).
  c. Cell fixation and EZH2 immunofluorescence staining.
    i. 24 h post cell seeding, remove cell culture media and wash the cell monolayer once with PBS Ca^2+^/Mg^2+^ pre-warmed at 37°C.
      ***Note:*** Calcium and magnesium support epithelial cell adhesion, and the pre-warmed buffer helps maintain attachment during washing and before fixation.
    ii. Fix the cells with 2.4% paraformaldehyde solution for 10 min at 20°C–25°C.
      **CRITICAL:** Both fixation temperature and duration strongly influence antigen preservation and staining quality.
    iii. Rinse the cells twice with PBS, incubate for 5 minutes at 20°C–25°C for each washing step.
      ***Note:*** PBS can be kept at 20°C–25°C for all following steps.
      **Pause point:** Fixed coverslips can be stored in PBS for a few days at 4°C.
      ***Optional:*** To prevent bacterial growth, supplement with Sodium Azide at a concentration of 0.05%.
    iv. Permeabilize cells with PBS containing 0.5% Triton X-100 for 10 min at 20°C–25°C.
      **CRITICAL:** Detergent concentration and permeabilization time determine antibody accessibility and signal quality.
    v. Rinse cells three times with PBS, incubating for 5 min at 20°C–25°C for each wash.
    vi. Block with 3% BSA diluted in PBS containing 0.5% Triton X-100 for at least 1 h at 20°C–25°C.
    vii. Prepare primary antibody staining solution by diluting the anti-EZH2 antibody 1:250 in blocking solution for immunofluorescence.
    viii. Incubate coverslips with the primary antibody solution overnight at 4°C.
      ***Note:*** To reduce antibody consumption, invert coverslips onto small droplets of antibody solution placed on Parafilm. We routinely use 80 μL antibody dilution for a 16 mm × 16 mm coverslip. Seal the incubation chamber with Parafilm to prevent evaporation.
      ***Optional:*** Add a piece of PBS-prewetted tissue to maintain humidity during overnight incubation.
    ix. Rinse cells three times with PBS, incubating for 5 min at 20°C–25°C for each wash.
    x. Prepare secondary antibody staining solution by diluting the goat anti-rabbit Alexa 488 1: 2500 in blocking solution for immunofluorescence. Include DAPI at 5 μg/mL to counterstain DNA.
      **CRITICAL:** Prepare the secondary antibody dilution shortly before use and keep protected from light to prevent photobleaching.
    xi. Incubate coverslips with the secondary antibody solution for 1 h at 20°C–25°C.
      **CRITICAL:** To prevent photobleaching, incubate the samples in the dark e.g. by covering the dish with aluminum foil.
    xii. Rinse cells three times with PBS, incubating for 5 min at 20°C–25°C for each wash.
    xiii. Mount coverslips in mounting medium (e.g., ProLong Gold) and allow curing overnight at 20°C–25°C before imaging.
    xiv. Image the stained samples on a confocal microscope such as the Zeiss LSM980 Airyscan or the Leica Stellarise8-DIVE.
      ***Note:*** Samples remain suitable for imaging for up to 2 weeks after fixation and staining. Store samples at all times protected from light to minimize fluorophore bleaching. Store the slides at 4°C, when not used for imaging.

### Cell sample preparation for Microscoop photolabeling

**Timing: 2–3 days**

In this step, we describe how to prepare fixed cell samples for Microscoop® photolabeling.

3. Seed cells for photolabeling.
  a. Seed approximately 4.5 × 10^5^ BoM-1833 cells in 1 mL of Complete cell culture medium supplemented with Penicillin-Streptomycin per one-well chamber slide to achieve around 80% confluency the next day at the time of cell fixing.
  b. Maintain the cells in a 37°C humidified incubator with 5% CO_2_ till the next day.

**CRITICAL:** The total number of slides needed for each project depends on the size and abundance of the target of interest. Troubleshooting 3.

***Note:*** For each biological replicate of in this EZH2 study, we used five chamber slides for photolabeling as photolabeled samples (PL) and five additional chamber slides as unlabeled controls (UL). While we have used BoM-1833 cells in this protocol, users can replicate the workflow in any cell line that displays PRC2 clusters visible. Adjustment in cell number for seeding may be needed to achieve roughly 80% confluency the next day at the time of cell fixing.

4. Fix, permeabilize and block cells.
  a. Incubate BoM-1833 cells with 2.4% paraformaldehyde under static conditions for 10 min at 20-25°C.
  b. Rinse the cells twice with 1× PBS.
  c. Permeabilize the cells with 1× PBS containing 0.5% Triton X-100 under static conditions for 10 min at 20-25°C.
  d. Wash the cells three times with 1× PBS.
  e. Block the cells with 3% BSA in 1× PBS containing 0.5% Triton X-100 on a seesaw shaker at 15 rpm for 1 h at 20°C–25°C.
5. Prepare samples for Microscoop® photolabeling by using SynLight-Rich™ Kit.
  a. Incubate the cells with 1 mL of Block 1 on a seesaw shaker at 15 rpm for 30 min at 20°C–25°C.
  b. Rinse the cells three times with 1× PBS containing 0.1% Triton X-100.
  c. Incubate the cells with 1 mL of Block 2 on a seesaw shaker at 15 rpm for 15 min.
  d. Rinse the cells three times with 1× PBS containing 0.1% Triton X-100.
6. Immunofluorescence staining targeting EZH2.
  ***Note:*** To ensure optimal staining consistency and reduce background noise, all antibody incubation and washing steps for fixed cell slides should be performed using a seesaw shaker set at 15 rpm. This gentle agitation ensures even distribution of reagents.
  a. Incubate the cells with rabbit anti-EZH2 antibody (1:500 dilution in 3% BSA in 1× PBS containing 0.1% Triton X-100) for 4 h at 20°C–25°C.
  b. Wash the cells three times with 1× PBS containing 0.1% Triton X-100 for 5 min each.
  c. Incubate the cells with anti-rabbit Alexa Fluor 647 secondary antibody (1:500 dilution in 3% BSA in 1× PBS containing 0.1% Triton X-100) for 2 h at 20°C–25°C in the dark.
  d. Wash the cells three times with 1× PBS containing 0.1% Triton X-100 for 5 min each.
  e. Rinse the cells three times with 1× PBS.
  f. Store the samples in 1× PBS at 4°C until use.
    **Pause point:** Fixed samples can be stored in 1× PBS for 1 to 2 weeks at 4°C. To extend the storage, storing samples in 1× PBS with sodium azide as preservative at a concentration of 0.02–0.1% at 4°C could further preserve the samples to up to 1 month.

### Microscoop image processing and photolabeling

**Timing: Steps 5 and 6: 1–2 h**

**Timing: Step 7: variable, 8–12 h per one-well chamber slide**

**Timing: Step 8: 1 day**

This step outlines the procedure for image-guided photolabeling of EZH2 clusters using Microscoop® and subsequent verification of biotinylation efficiency. The general protocol also applies to other targets of interest.

**CRITICAL:** The Photolabel reagent is sensitive to light. When using the reagent and operating photolabeling procedures, one should perform under a light-controlled environment. Ensure the lighting is at a wavelength of > 500 nm to prevent undesirable reactions. In our laboratory, procedures were performed in a dedicated red-light room.

7. Take two EZH2-stained chamber slides, one serving as the photolabeled (PL) sample and the other as the unlabeled (UL) sample. Add 1 mL Labeling Reagent to each slide.

**CRITICAL:** Do not use DAPI (365 nm) or brightfield imaging once the Labeling Reagent has been added to the samples.

**Figure 3:**
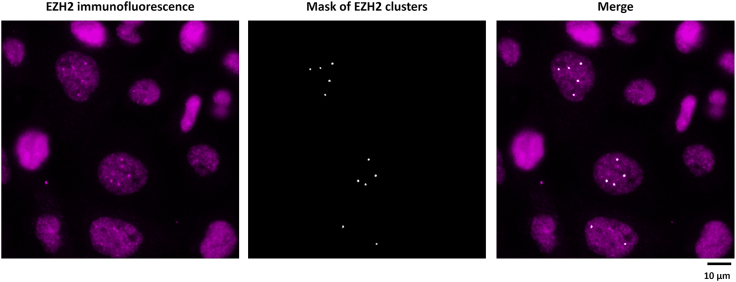
Microscoop® Mint–based ROI recognition and photolabeling of EZH2 clusters A photolabeling mask was generated from the immunostaining signal of EZH2 clusters (magenta) in BoM-1833 cells using image-processing functions. The images shown were acquired on the Microscoop® system during mask preparation. Scale bar: 10 μm.

8. Place one slide onto the stage of Microscoop for photolabeling (PL).
9. Control the system with the software, Autoscoop.
  ***Note:*** Within Autoscoop, there are 3 modules to use in the following orders: (1) Imaging, (2) Pattern Generation and (3) Photolabeling (Figure 2).
  a. Acquire a fluorescent image of the target protein or region of interest (ROI) in the Imaging module.
  b. Load the image in the Pattern Generation module.
  c. Generate an image processing mask targeting the ROI by selecting combinations of pre-installed image processing functions.
    ***Note:*** Selection of algorithms, e.g. intensity filters, size filters etc., is based on the image features of the ROI.
  d. Use the image-processing algorithm (Particle Extraction) to generate a mask that identifies EZH2-positive clusters (Figure 3).

**Figure 2:**
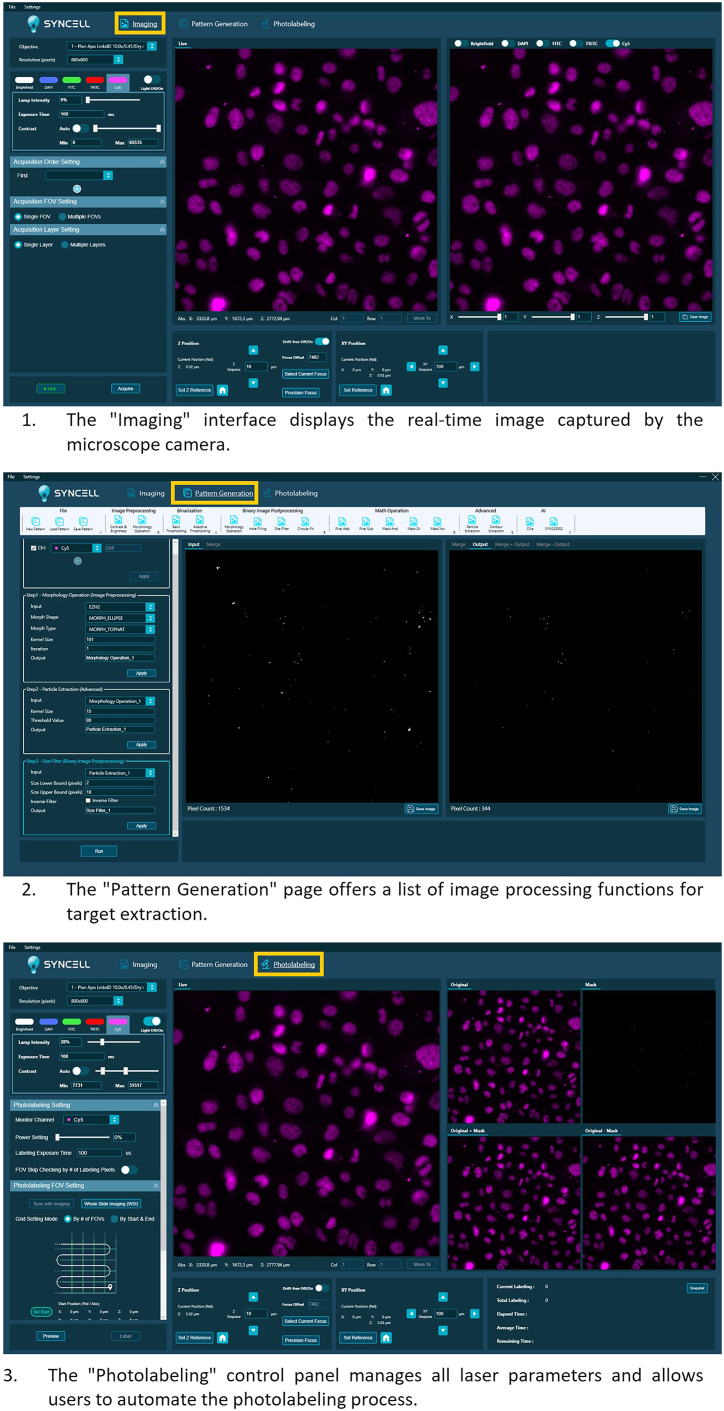
Core Workflow Modules of the Microscoop® System The figure displays the three primary user interfaces (UIs) used for experimental execution. **Imaging**: Provides real-time images from the microscope camera. Imaging parameters, including channel selection, lamp intensity, and exposure time, can be adjusted in the left panel. **Pattern Generation**: The upper toolbar contains image-processing functions used to define targets for labeling, while the left panel displays the masking procedures. The central workspace displays the acquired images together with their corresponding masks and calculates the pixel count for each image. **Photolabeling**: Serves as the central control panel for managing laser parameters (power and labeling time) and automating the labeling sequence. During photolabeling, the pixel count and labeling duration are recorded in the bottom-right panel.
10. Set the photolabeling laser parameters according to the Synlight-Rich kit recommendations.

**CRITICAL:** Keep the laser power ≤ 200 mW and the labeling exposure time ≤ 1,000 μs per pixel.

***Note:*** For BoM-1833 cell EZH2 clusters, the photolabeling conditions were set to a laser power of 200 mW and a labeling exposure time of 1,000 μs per pixel. Under the 40×/0.95 NA objective, the BoM-1833 cell nucleus has a diameter of approximately 10–15 μm, and EZH2 clusters range from 0.8–1.2 μm in diameter, typically occupying about 0.3%–1.4% of the total nuclear area.

11. Initiate photolabeling of the PL in the Autoscoop to automatically label the masked regions across the entire chamber slide.

**CRITICAL:** Keep the other slide in a covered container near the microscope to protect it from light and ensure it undergoes similar conditions (e.g., similar reagent incubation time). This will serve as unlabeled control (UL).

12. Upon completion of Microscoop® photolabeling, quench the reaction.
  a. Wash the PL and UL cell samples three times with 1 mL of Quencher on a seesaw shaker at 15 rpm for 5 min each.
  b. Wash the cells thoroughly in 1× PBS containing 0.1% Triton X-100 overnight on a seesaw shaker at 15 rpm at 4°C.
  c. Store the samples in 1× PBS at 4°C until all samples have been photolabeled and are ready to proceed to the subsequent immunoprecipitation step.

***Note:*** Repeat steps 5–8 until all five photolabeled samples (PL) and five corresponding unlabeled controls (UL) have been processed.

**CRITICAL:** When performing pilot tests or running the procedure for the first time, verify biotinylation efficiency prior to proteomic processing.

For efficiency assessment, stain a small portion of the PL regions with the Verification Buffer prepared from the SynLight-Rich™ kit (step 10). Samples treated with Verification Buffer are unsuitable for downstream proteomic analysis.

Reserve the majority of sample for subsequent enrichment and LC–MS/MS analysis (proceed to step 11).

13. Measure biotinylation efficiency.
  a. Incubate the cells with 1 mL of Verification Buffer on a seesaw shaker at 15 rpm for 1 h in the dark at 20-25°C.
  b. Wash the cells three times with 1× PBS containing 0.1% Triton X-100 on a seesaw shaker for 5 min each.
  c. Rinse the cells three times with 1× PBS.
  d. Use Microscoop with the FITC channel (490 nm) for epifluorescence imaging.
  e. Analyze photolabeling efficiency by calculating the signal-to-noise (S/N) ratio using ImageJ.
    i. Drag the image of PL samples stained with Verification Buffer into ImageJ.
    ii. Click “Image > Adjust > Threshold”. Adjust the Maximum slider to its maximum value and the Minimum slider until the ROIs are properly highlighted in red.
    iii. Click “Analyze > Measure” to record the mean intensity of the labeled ROIs.
    iv. Determine the threshold for Blank: set the Minimum slider to 0 and adjust the Maximum slider until the cell-free areas are properly highlighted in red.
    v. Click “Analyze > Measure” to record the mean intensity of the Blank.
    vi. Determine the threshold for the unlabeled intracellular region (between Blank and labeled ROI): set the Minimum slider to MaxThr of Blank and the Maximum slider to MinThr of labeled ROI.
    vii. Click “Analyze > Measure” to record the mean intensity of the unlabeled intracellular region.
    viii. Calculate photolabeling efficiency using the following formula:
         (I represents the mean intensity).
      $$\frac{S}{N}=\frac{I_{labeled\;ROI}-I_{Blank}}{I_{unlabeled\;intracellular\;region}-I_{Blank}}$$
      ***Note:*** The recommended acceptance criterion for the S/N ratio is > 8.0.

### Sample immunoprecipitation for mass spectrometry

**Timing: 3 days**

This step describes how to process samples using the Synpull™ reagent kit, covering the lysis of photolabeled (PL) and control (UL) cells, followed by enrichment of biotinylated proteins with streptavidin beads. The procedure also includes on-bead digestion and peptide preparation for downstream mass spectrometry analysis.

***Note:*** Use low retention tips for all procedures to minimize sample loss.

14. Harvest the photolabeled and unlabeled cells.
  a. Scrape the cells in Buffer A. Scrape and transfer them into separate tubes, collecting all 5 PL cells samples in one tube (PL) and all 5 UL cell samples in another (UL).
  b. Centrifuge the samples at 5000 × *g* for 3 min at 4°C and retain the cell pellet with approximately 100 μL remaining buffer.
15. Perform sample lysis.
  a. Add 60 μL Lysis Buffer to each pellet and lyse the cells by probe sonication for 60 cycles (1s on/2s off) at 30% amplitude (Qsonica, Q125, maximum power output 125 W).
  b. Spin down the lysates and heat them at 99°C for 1 h.
  c. Cool the samples and add 15 μL of Solution D (Lyse 3).
  d. Mix thoroughly and incubate at 20°C–25°C for 30 min in the dark.
  e. Centrifuge at 16000 × *g* for 20 min at 4°C and transfer the supernatants into new tubes.

***Note:*** If low protein yield is observed, refer to Troubleshooting 4.

**Pause point:** At this point, the lysates can be stored at 4°C if the subsequent pulldown can be performed within 24 h. If longer time is needed before proceeding to the next step, they can be stored at −20°C for up to 1 month.

16. Determine protein concentration using the Pierce 660 nm protein assay (IDCR method; Thermo Fisher Scientific, Cat#22662 & Cat#22663).
17. Perform immunoprecipitation of biotinylated proteins.
  a. Use 200 μg protein each for PL and UL into the CA (Proteomics tube) as input amount for pull-down.
    ***Note:*** Typically, 40–60 μg of total protein can be extracted from a single-well chamber slide under our experimental conditions. In this study, five slides were pooled per biological replicate, yielding approximately 200 μg of total protein for pull-down. Lower input amounts may be feasible depending on the size and abundance of the photolabeling target as well as the mass spectrometer sensitivity. See troubleshooting 3 for further discussion on sample input optimization.
  b. Dilute each sample 5-fold with Solution E.
  c. Wash the streptavidin magnetic beads F (Pull) before use.
    i. Add 200 μL of E (Dilute) into F (Pull) and vortex the tubes at 1000 rpm for 5 min at 20°C–25°C.
    ii. Briefly centrifuge the F (Pull) tube to collect all contents at the bottom and place it on the magnetic rack for 2 min to ensure complete collection of the beads against the side of the tubes.
    iii. Remove the supernatant carefully and gently resuspend the beads with 80 μL of E (Dilute).
      ***Note:*** Other commercially available streptavidin magnetic beads, for example, the NanoLINK Streptavidin Magnetic Beads, may also be suitable for the pull down, provided that it is streptavidin conjugated and magnetic-based beads. However, since binding capacity and bead surface may differ, users may need to optimize bead amount and incubation conditions based on the manufacturer’s protocol of the corresponding beads.
      ***Note:*** Unless otherwise specified, the brief spin-down in Step 17 following vortexing is intended solely to collect liquid from the tube walls and cap. A centrifugal force of 1000 × *g* for less than 1 minute (or a brief pulse) is sufficient for this purpose. This ensures maximum sample recovery and maintains volume consistency throughout the immunoprecipitation process.
  d. Add streptavidin magnetic beads to the samples and incubate with rotation for 1 h at 20°C–25°C to pull down biotinylated proteins.
    ***Note:*** The protein-to-bead ratio (μg/μL) and the incubation duration may require optimization based on different targets. Initial testing can be performed using 200–400 μg of protein with 10 μL of beads and an incubation period of 1 hr.
    ***Note:*** Store the unused beads F (Pull) at 4°C. Avoid freezing the beads as this may potentially damage its chemical properties.
  e. Spin down and place the tubes on the magnetic rack for 2 min. Then, carefully discard the supernatants.
    **CRITICAL:** At each magnetic separation step, ensure that the beads are fully adhered to the magnetic surface before removing the supernatant, and discard the supernatant carefully to avoid disturbing or losing the beads.
  f. Wash the protein-bound beads four times using the designated wash buffers.
    i. Add 1 mL of G (Wash 1) and vortex the tubes at 1000 rpm for 5 min.
    ii. Spin down and place the tubes on the magnetic rack for 2 min.
    iii. Carefully discard the supernatant.
    iv. Repeat steps i–iii using H (Wash 2).
    v. Repeat steps i–iii using I (Wash 3).
    vi. Add 1 mL of J (Wash 4) and vortex the tubes at 1000 rpm for 5 min.
    vii. Centrifuge at 5000 × *g* for 2 min.
    viii. Place the tubes on the magnetic rack for 2 min.
    ix. Carefully discard the supernatant.
  g. Resuspend the beads in 100 μL of J (Wash 4) and sonicate in an ultrasonic water bath for 10 s.
  h. Spin down and place the tubes on the magnetic rack for 2 min. Then, carefully discard the supernatants.
  i. Repeat steps g–h until there is a noticeable thin and sticky appearance of the beads against the magnetic rack, indicating successful removal of detergents.
18. Conduct on-bead digestion.
  a. Resuspend the beads in 20 μL of Solution K (Digest).
  b. Incubate the samples at 37°C for 2 h using an Intelli-Mixer™ (ELMI, RM-2S) in uu mode (small amplitude mixing) at a 35° angle with an intensity of 35.
    **CRITICAL:** Ensure that the beads remain well mixed (with no visible precipitates) and avoid splashing during the process. When using alternative mixing devices, choose a gentle rocking or vibration mode if available (or low orbital amplitude) and set RPM around ∼30–40 as a starting point. Adjust until no splashing is observed.
    ***Note:*** Trypsin protease from Thermo Fisher Scientific, Waters or other vendors may also be used, provided that it is MS-grade. However, as enzyme activity and digestion efficiency may vary, digestion conditions (e.g., enzyme-to-substrate ratio or incubation time) may require optimization based on manufacturer’s protocol of the corresponding enzyme.
  c. Spin down and place the tubes on the magnetic rack for 30 s. Transfer the supernatants to new proteomics tubes.
  d. Continue incubation at 37°C for 16 h with 35° shaking.
19. Perform peptide desalting prior to LC-MS/MS analysis.
  a. Add 2 μL of Solution L (Stop) to each sample and mix thoroughly to stop the reaction.
  b. Take 0.5 μL of each sample and verify that the pH is below 4 using pH test strips.
    ***Note:*** If there is any uncertainty regarding the pH value, L (Stop) can be added to a maximum of 4 μL.
  c. Prepare the desalting buffers for each sample:
    i. Add 100 μL of Desalt 1 buffer to a new 1.5-mL tube.
    ii. Add 200 μL of Desalt 2 buffer to a new 1.5-mL tube.
    iii. Add 20 μL of Desalt 3 buffer to a new CA proteomics tube labeled with the specific sample name.
  d. Start the desalting procedure using ZipTip C18 resin:
    i. Attach a ZipTip to a P20 Pipetman.
    ii. Activation: Place the ZipTip in Desalt 1 buffer. Aspirate 20 μL of Desalt 1 buffer and dispense the solution as waste. Repeat 5 times.
    iii. Equilibrium: Place the ZipTip in Desalt 2 buffer. Aspirate 20 μL of Desalt 2 buffer and dispense the solution as waste. Repeat 5 times.
    iv. Binding: Place the ZipTip in the sample and perform 30 aspirate–dispense cycles to ensure thorough binding of peptides to the ZipTip.
    v. Wash: Place the same ZipTip in Desalt 2 buffer. Aspirate 20 μL of Desalt 2 buffer and dispense the solution as waste. Repeat 5 times.
    vi. Elution: Place the same ZipTip in Desalt 3 buffer and perform 5 aspirate–dispense cycles to elute all peptides into the tube. The peptides are now desalted.
    vii. Discard the used ZipTip after the elution step.
  e. To desalt additional samples, repeat Step d using a new ZipTip.
20. Concentrate the desalted peptides in a SpeedVac at 20°C–25°C until completely dry (∼2 h).

**Pause point:** The dried samples can be preserved at −20°C for up to 1 month until LC-MS/MS analysis.

### Detection of immunoprecipitated products by data-dependent acquisition mass spectrometry

**Timing: 1–2 days**

This step describes how to analyze desalted peptides by liquid chromatography-tandem mass spectrometry (LC-MS/MS) using DDA to identify pull-down biotinylated proteins. It covers peptide resuspension, NanoLC setup, and detailed MS/MS acquisition parameters for Orbitrap Fusion Lumos.

21. Reconstitute desalted peptides in 12 μL of 0.1% formic acid (FA) in LC-MS–grade water by 20–30 dispense–aspirate cycles.

***Note:*** The peptides after desalting were not quantified prior to MS analysis to avoid potential sample loss given the low amount of starting material. Instead, the total peptides yielded from each preparation was resuspended in 12 μL of 0.1% formic acid (FA) in this study. For the LC-MS/MS analysis, 5 μL of this suspension was injected in duplicate (technical replicates). An equal volume of injection across all photolabeled (PL) and Unlabeled (UL) groups were used.

***Note:*** The peptide injection volume for LC–MS/MS should be adjusted according to the loading capacity of the LC column to prevent column overloading and potential clogging.

22. Centrifuge the reconstituted peptides at 16000 × *g* for 2 min at 20°C–25°C, then transfer the supernatants to LC-MS/MS sample vials.

***Note:*** Ensure that no air bubbles remain at the vial bottom.

23. Load 5-μL of samples for 2 times into the UltiMate 3000 RSLCnano system coupled to an Orbitrap Fusion Lumos mass spectrometer with PepMap 100 C18 HPLC column (2 μm, 100 Å, 75 μm × 25 cm) at 300 nL/min using the following gradient:

**Table:** 

Time (min)	Solvent a (0.1% FA/Water)	Solvent B (0.1% FA/Acetonitrile)
0	98%	2%
2	96%	4%
85	80%	20%
108	62%	38%
109	5%	95%
113	5%	95%
114	98%	2%
120	98%	2%

24. Operate the Orbitrap Fusion Lumos instrument in higher-energy collisional dissociation (HCD) mode for peptide fragmentation with 30% collision energy.
25. MS1 survey scan:
  a. Perform survey scans on the Orbitrap mass analyzer at a resolution of 120,000 with an automatic gain control (AGC) target of 4 × 10^5^ and a maximum injection time of 50 ms.
  b. Acquire ions across an m/z range of 375–1500.
26. Precursor selection:
  a. Use Top-Speed DDA to select precursors for MS/MS fragmentation.
  b. Include precursor ions with charge states of 2+, 3+, or 4–7+, and exclude ions with unassigned charge states, 1+, or > 8+.
  c. Set quadrupole isolation windows to 1.2 Th for 2+ ions, 0.7 Th for 3+ ions, and 0.4 Th for 4–7+ ions.
  d. Apply dynamic exclusion for 60 s with a mass tolerance of ±10 ppm.
27. MS/MS acquisition:
  a. Acquire fragment ion spectra in the Orbitrap at a resolution of 30,000.
  b. Set the AGC target to 5 × 10^4^ and the maximum injection time to 54 ms.
  c. Use a maximum cycle time of 3 s.

### Protein identification and label-free quantification

**Timing: 1–2 days**

In this section, raw DDA files are processed in Proteome Discoverer for peptide/protein identification and label-free quantification, with defined search settings, FDR thresholds, normalization steps, and criteria applied for downstream analysis.

28. Proteome Discoverer-based data processing and database search:
  a. Process raw data from the same two-photon illumination batch using Proteome Discoverer (version 2.4.1.15) and identify peptides/proteins with Sequest HT against the UniProtKB/Swiss-Prot human database (version 2020.03).
  b. Configure the database search using the following parameters:
    i. Enzyme: trypsin, allowing up to three missed cleavages.
    ii. Precursor mass tolerance: 10 ppm.
    iii. Fragment mass tolerance: 0.05 Da.
    iv. Static modification: carbamidomethylation on Cys (+57.0215 Da).
    v. Dynamic modifications:
         Deamidation on Asn and Gln (+0.9840 Da),
         Oxidation on Met (+15.9949 Da),
         Acetylation on protein N-termini (+42.0106 Da).
    vi. Minimum peptide length: 6 amino acids.
  c. Set the false discovery rate (FDR) threshold to 1% at both the peptide and protein levels.
29. Label-free quantification using Proteome Discoverer:
  a. Perform retention-time alignment using a chromatographic time window of 20 min.
  b. Normalize peptide-level intensities to the total peptide ion signal of each LC-MS run.
  c. Derive protein-level abundance by summing the normalized intensities of the top three most intense unique peptides assigned to each protein.

***Note:*** Other MS search and analysis programs such as MaxQuant can also be used for analysis.

30. Data visualization and ROI protein derivation.
  a. Compute the protein abundance ratio of PL to UL samples using a t-test.
  b. Apply a log_2_ transformation to the fold change (PL/UL) and a −log_10_ transformation to the p-value for each protein.
  c. Generate a volcano plot using log_2_(PL/UL) as the x-axis and −log_10_(p-value) as the y-axis.
  d. To derive putative ROI proteins, apply the following filters to the protein list:
    i. log_2_(fold change) ≥ 0.58 (corresponding to fold change ≥ 1.5).
    ii. −log_10_(p-value) ≥ 1.3 (corresponding to p ≤ 0.05).
    iii. Number of unique peptides ≥ 2.
  e. The filtered proteins can be further prioritized by their fold change, with a higher ratio indicating greater confidence in successful downstream validation.

***Note:*** If LC–MS/MS results show low sensitivity, refer to Troubleshooting 5.

***Note:*** If LC–MS/MS results show low specificity of ROI proteins or failure to detect target proteins, refer to Troubleshooting 6.

## Expected outcomes

**Figure 4:**
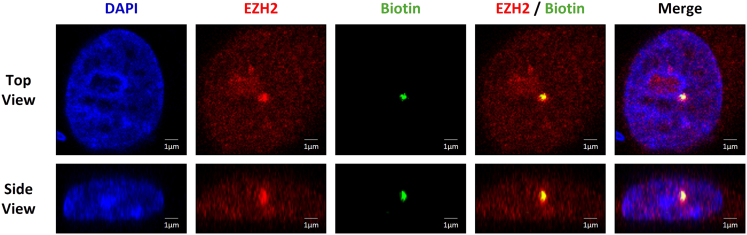
Confocal validation of spatially restricted photo-biotinylation within EZH2 clusters BoM-1833 cells were immunostained for endogenous EZH2 (red) and photolabeled using the Microscoop® platform. Photo-biotinylated proteins were detected with Dy488-NeutrAvidin (green), and nuclei were counterstained with DAPI. A single focal plane and an axial side view from confocal imaging illustrate biotinylation confined to EZH2 clusters. Scale bar: 1 μm.

Using Microscoop®, an image processing mask specifically targeting EZH2 clusters can be generated using the pre-installed image processing algorithms in the Pattern Generation Module inside the Autoscoop Software (Figures 2 and 3). With a good calibrated Microscoop®, a precise masking and photolabeling can be achieved. This can be confirmed by confocal microscopy of the co-staining of the target primary antibody and biotin using the Verify reagent in the Synlight Rich kit after photolabeling. A precise biotinylation shows co-localization of biotin signal within the ROI. In this study, the BoM-1833 cells were stained for endogenous EZH2 (red) and biotin (green). Representative confocal images (Figure 4) show that the photo-biotinylated regions colocalize with the EZH2 clusters, confirming successful and spatially restricted photolabeling.

**Figure 5:**
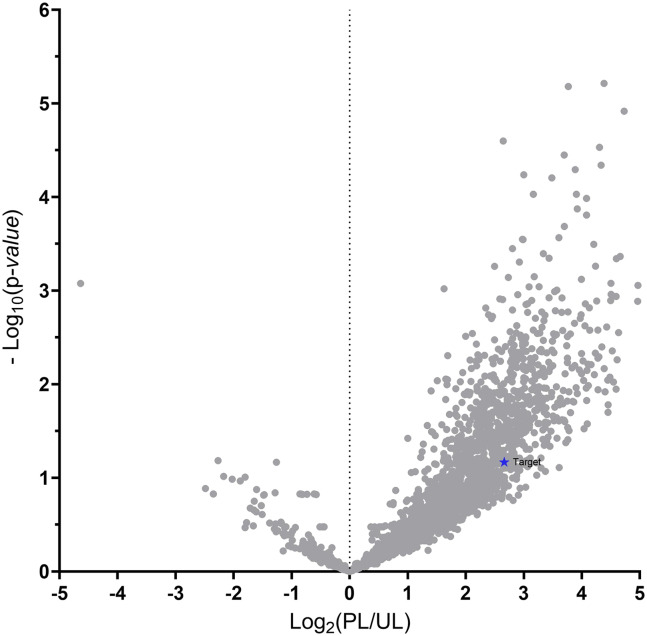
An example of volcano plot summarizing MS data generated from optoproteomics workflow After LC–MS/MS analysis, photolabeled samples (PL) were compared with corresponding non-illuminated/unlabeled controls (UL) to generate a volcano plot, with x axis being fold change difference between PL and UL, and y-axis being p-value. A right-skewed volcano plot and an identification of the photolabeling target is expected. The dataset used for preparing this figure has been previously published.^1^

Following LC–MS/MS analysis, a volcano plot is generated to visualize the data distribution (Figure 5). A good enrichment of proteins in the PL group versus the UL group is indicated by a right-skewed volcano plot using fold change of PL/UL as x-axis. The expected target protein of interest should be in the top-right region of the volcano plot, ideally with a PL/UL fold change ≥ 1.5.

## Limitations

The basic concept of the opto-proteomic workflow with Synlight Rich reagents is to use light to activate a biotin-based reagent, thereby causing its covalent binding to amino acid residues in proteins, i.e., biotinylation. The illumination pattern is defined by image-based processing, allowing spatially controlled labeling. However, as biotinylation is directed by light, the effective labeling radius, i.e., the labeling resolution is determined by the laser beam size at the focal plane of the objective. Using the 40X air objective (NA 0.95) and a two-photon illumination in the Microscoop®, the photolabeling resolution of Synlight Rich photolabel reagent is approximately 0.35 μm in xy plane and 1.5 μm along the z axis. This sets a fundamental limit on the minimum size of targetable structures and makes the approach suitable only for regions of interest (ROIs) larger than this resolution. Targeting structures below this limit may result in false-positive labeling as the volume of regions being biotinylated would exceed the size of the ROIs.

In addition, recovery of biotinylated proteins relies on streptavidin-based pull-down. Non-specific binding of proteins to the pull-down matrix can introduce false-positive identifications. Therefore, appropriate experimental controls, particularly unlabeled controls, are essential to distinguish true signal from background. Thorough and careful washing during the pull-down step is also critical to minimize non-specific interactions and improve data specificity.

## Troubleshooting

### Problem 1

Weak or absent EZH2 immunofluorescence signal (Steps 1–9).

### Potential solution

EZH2 is a nuclear protein that is strongly associated with chromatin. Suboptimal permeabilization, caused, for example, by high cell confluency on glass coverslips or an insufficient detergent concentration, can limit epitope accessibility and result in weak or absent staining.

• Ensure cells are no more than ∼70% confluent at the time of fixation.
• Increase the Triton X-100 concentration in the permeabilization buffer.
• Extend the permeabilization duration.

### Problem 2

Low signal-to-background ratio in EZH2 immunofluorescence; PRC2 bodies poorly distinguishable (Steps 1–9).

### Potential solution

• Optimize antibody concentrations: Titrate the EZH2 primary (and secondary) to the lowest concentration that preserves clear nuclear/cluster signal.
• Improve blocking: Increase BSA concentration during incubations, extend blocking time.
• Increase wash stringency: Add additional washes, extend wash time, and increase Triton X-100 concentration in wash buffer to reduce non-specific binding.
• Validate secondary specificity: Use highly cross-adsorbed secondaries; include a “secondary-only” control to assess background.
• Refine immunofluorescence conditions: We also observed good performance using the following alternative immunofluorescence procedure: cells were fixed in 3% PFA + 2% sucrose for 10 min at 20 – 25°C, followed by permeabilization for 5 min on ice in Triton buffer (0.5% Triton X-100, 20 mM HEPES, pH 7.4, 50 mM NaCl, 3 mM MgCl_2_, 300 mM sucrose). All subsequent staining and wash buffers contained 0.1% Tween-20.
• Adjust imaging settings: Reduce laser power/gain and avoid saturation; acquire all conditions with identical settings.

### Problem 3

Underestimating sample number for an optoproteomics experiment (Steps 3, 17).

### Potential solution

The total amount of sample required therefore depends largely on the size and abundance of the target structure, which determines how many pixels can be labeled per slide. The number of photolabeled pixels in a field of view can be estimated during mask generation, a module available in the Microscoop software. A critical parameter for a successful Microscoop–MS experiment is to achieve a sufficient number of photolabeled pixels. In practice, a minimum of approximately 10^7^ photolabeled pixels per biological replicate is recommended to enable robust protein identification by LC–MS/MS. Small or low-abundance targets may require pooling a larger number of slides to reach the recommended pixel count.

In our workflow, a single well of a chamber slide typically yields ∼40–60 μg of total protein lysate. Pooling material from five slides per biological replicate results in approximately ∼200 μg total protein input for the pull-down step. This amount was selected primarily as a practical precaution to compensate for potential material loss during enrichment, rather than as a strict requirement of the Microscoop labeling process. Using ∼200 μg input protein have rendered robust protein identification on an Orbitrap Fusion Lumos mass spectrometer. Lower protein input may be feasible when using higher-sensitivity instruments, such as the Orbitrap Astral (Thermo Fisher Scientific).

### Problem 4

Low protein yield from cell lysates (Step 15).

### Potential solution

Low protein recovery may result from aggregation of the cell pellet, which can be addressed by extending the sonication time to reduce the amount of visible pellet. Conversely, overly strong sonication may cause the sample to splash, leading to loss of material and reduced yield. In such cases, use an ultrasonic water bath instead for gentler disruption.

### Problem 5

Low protein sensitivity in LC-MS/MS analysis (Step 30).

### Potential solution

• Missed cleavages during digestion (>25%).

 Confirm that K (Digest) has been stored at −20°C and has not been freeze–thawed more than three times. Ensure that the sample is incubated with Solution K (Digest) for at least 16 h to achieve complete digestion.

• Bead precipitation during on-bead digestion.

 Ensure that the beads remain well mixed and fully resuspended in Solution K (Digest) during the digestion process. Avoid splashing, which can lead to loss of material.

• Incorrect buffer conditions or order during the desalting step.

 After adding L (Stop), verify that the sample pH is below 4. Confirm that the buffers and the sequence of steps in the desalting procedure are applied correctly.

### Problem 6

Low specificity of ROI proteins or failure to identify target proteins (Step 30).

### Potential solution

Low specificity of ROI proteins may occur when labeling efficiency is insufficient. Prepare new samples and repeat the labeling process to achieve higher efficiency (see Note about Photolabeling Efficiency Measurement). Ensure that all notes and critical steps described in the protocol have been followed.

Failure to detect the target or well-known proteins may be due to their inherently low abundance. Increase the number of samples or slides used for LC–MS/MS to boost detection sensitivity. Alternatively, consider using a data-independent acquisition (DIA) approach to improve the identification of low-abundance proteins.

## Resource availability

### Lead contact

Further information and requests for resources and reagents should be directed to and will be fulfilled by the lead contact, Cristiana Lungu (cristiana.lungu@izi.uni-stuttgart.de).

### Technical contact

Technical questions on executing this protocol should be directed to and will be answered by the technical contacts, Weng Man Chong (tina.chong@syncell.com) or Jung-Chi Liao (jcliao@syncell.com).

### Materials availability

This study did not generate new unique reagents.

### Data and code availability

This study did not generate/analyze new code or dataset. The optoproteomics dataset for endogenous EZH2 clusters in BoM-1833 cells was already described in the study by Pelzer et al.^1^

## Acknowledgments

The work of C.L. was supported through a grant by the Baden-Wuerttemberg Ministry of Science, Research and Arts. C.L. gratefully acknowledges the Technology Platform “Cellular Analytics” of the Stuttgart Research Center Systems Biology for their support with imaging. C.L. thanks Peter Teufel for assistance with the optimization of EZH2 immunofluorescence. We thank Jasmine and Chia-Min Liao for editorial assistance and help with figure preparation. Step 1 of the graphical abstract was generated using Biorender.

## Author contributions

Conceptualization, C.L.; methodology, C.L. and W.M.C.; investigation, C.L., Y.-C.P., and H.-J.C.; writing – original draft, C.L., Y.-C.P., and H.-J.C.; writing – review and editing, C.L. and W.M.C.; funding acquisition, C.L.; supervision, C.L. and J.-C.L.; project administration, C.L.

## Declaration of interests

J.-C.L., W.M.C., Y.-C.P., and H.-J.C. are employees and shareholders of Syncell Inc. J.-C.L. is the founder and the CEO of the company. W.M.C. is the senior application manager in the Applications Department, Y.-C.P. is the staff scientist in the Applications Department, and H.-J.C. is the scientific manager in the Biochemistry Research Department. In addition, J.-C.L. and W.M.C. are inventors on patent applications related to the Microscoop technology, and J.-C.L., W.M.C., and H.-J.C. are inventors on a patent application related to the Synlight-Rich kit, which are related to the work described in this article.

## Declaration of generative AI and AI-assisted technologies in the writing process

During the preparation of this work, C.L. used ChatGPT 5.1 in order to improve readability and the language of the manuscript draft. After using this tool, the edited content was reviewed by C.L., who takes full responsibility for the final publication.
